# Scattering of Light by Colloidal Aluminosilicate Particles Produces the Unusual Sky-Blue Color of Río Celeste (Tenorio Volcano Complex, Costa Rica)

**DOI:** 10.1371/journal.pone.0075165

**Published:** 2013-09-18

**Authors:** Erick Castellón, María Martínez, Sergio Madrigal-Carballo, María Laura Arias, William E. Vargas, Max Chavarría

**Affiliations:** 1 Escuela de Química, Universidad de Costa Rica, San José, Costa Rica; 2 Observatorio Vulcanológico y Sismológico de Costa Rica, Universidad Nacional (OVSICORI-UNA), Heredia, Costa Rica; 3 Laboratorio de Polímeros, Escuela de Química, Universidad Nacional, Heredia, Costa Rica; 4 Facultad de Microbiología, Universidad de Costa Rica, San José, Costa Rica; 5 Escuela de Física and Centro de Investigación en Ciencia e Ingeniería de Materiales, Universidad de Costa Rica, San José, Costa Rica; 6 Centro de Investigaciones en Productos Naturales (CIPRONA), Universidad de Costa Rica, San José, Costa Rica; University of Zurich, Switzerland

## Abstract

Río Celeste (Sky-Blue River) in Tenorio National Park (Costa Rica), a river that derives from the confluence and mixing of two colorless streams—Río Buenavista (Buenavista River) and Quebrada Agria (Sour Creek)—is renowned in Costa Rica because it presents an atypical intense sky-blue color. Although various explanations have been proposed for this unusual hue of Río Celeste, no exhaustive tests have been undertaken; the reasons hence remain unclear. To understand this color phenomenon, we examined the physico-chemical properties of Río Celeste and of the two streams from which it is derived. Chemical analysis of those streams with ion-exchange chromatography (IC) and inductively coupled plasma atomic emission spectroscopy (ICP-OES) made us discard the hypothesis that the origin of the hue is due to colored chemical species. Our tests revealed that the origin of this coloration phenomenon is physical, due to suspended aluminosilicate particles (with diameters distributed around 566 nm according to a lognormal distribution) that produce Mie scattering. The color originates after mixing of two colorless streams because of the enlargement (by aggregation) of suspended aluminosilicate particles in the Río Buenavista stream due to a decrease of pH on mixing with the acidic Quebrada Agria. We postulate a chemical mechanism for this process, supported by experimental evidence of dynamic light scattering (DLS), zeta potential measurements, X-ray diffraction and scanning electron microscopy (SEM) with energy-dispersive spectra (EDS). Theoretical modeling of the Mie scattering yielded a strong coincidence between the observed color and the simulated one.

## Introduction

**Figure 1:**
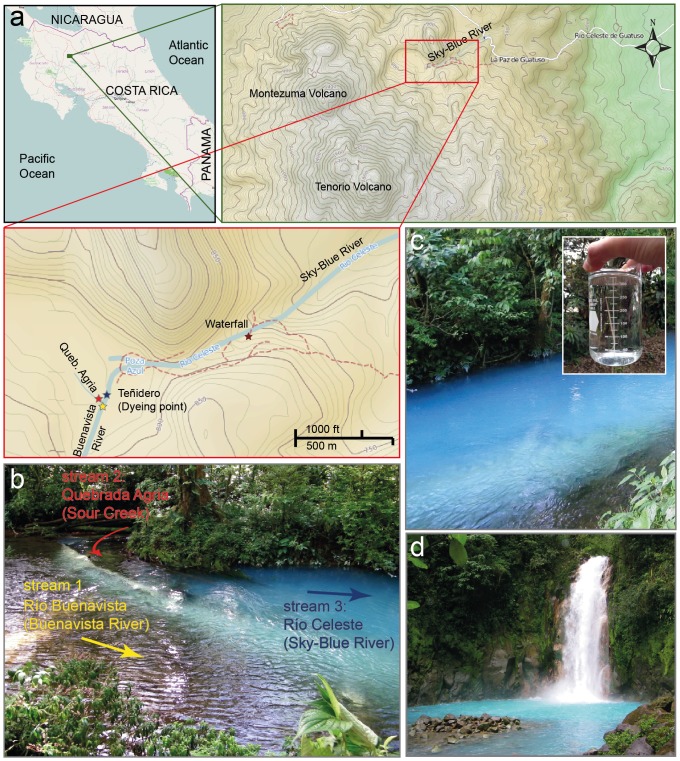
Location of Río Celeste and the dye point. (**a**) Río Celeste is located in Costa Rica within the Tenorio Volcano National Park (Alajuela, Guatuso, 10°42′02.5″ N, 84°59′49.3″ W). Maps taken from OpenStreetMaps (http://www.openstreetmap.org/). (**b**) As seen in the picture, the sky-blue color of Río Celeste originates from the mixing of two transparent streams (Río Buenavista and Quebrada Agria) at a point known as “Teñidero” (dye point). That characteristic makes a remarkable difference from other blue waters of thermal origin. (**c**) The intense sky-blue color appears immediately after the dye point. The sky-blue color is maintained just in the river: a water sample (see box in figure) outside the stream is completely colorless. (**d**) The river maintains its peculiar coloration over a distance more than 14 km including at the waterfall Río Celeste.

Costa Rica is a country of which volcanic activity has decisively influenced its formation. As part of the circum-Pacific ring of fire, over 400 volcanic foci have been recognized in the country, but only about 20 of them have significant sizes [1], [2]. Costa Rica has four mountain ranges, which are, from NW to SE, Guanacaste, Tilarán, Central Volcanic and Talamanca, but most volcanoes are located in the Guanacaste and central volcanic ranges [1]–[5]. Located in Guanacaste, Tenorio is a complex basaltic-andesitic volcanic massif consisting of a cluster of volcanic edificies aligned NNW-ESE. It is situated on the southeastern edge of the Guanacaste volcanic range, between Miravalles and Arenal volcanoes; the elevation of its summit is 1916 m above sea level [1], [2]. The Tenorio complex has numerous manifestations of hydrothermal activity and diffuse emission of gas at its NE end [6], [7]. One of the most conspicuous features is the existence of a sky-blue river known as Río Celeste (Alajuela, Guatuso, 10°42′02.5″ N, 84°59′49.3″ W), which is an important tourist attraction in Costa Rica with about 20,000 visitors annually (Fig. 1a).

An interesting aspect of the origin of the color of Río Celeste is that the sky-blue water occurs on the mixing of two colorless streams at a point known as Teñidero (dye point), as shown in figures 1a and 1b. This characteristic is remarkably different from other blue waters of thermal origin [8]–[15]. The confluence and mixing of Río Buenavista and Quebrada Agria originates Río Celeste (Fig. 1b). Upon the mixing of these streams, various colors, including sky-blue and cyan are generated. It is also possible to observe in the bottom of the river the presence of a sedimented white material. The river maintains its peculiar color over a distance of about 14 km, including a waterfall (Figs. 1c and 1d) and a blue lagoon. An inspection of a water sample of Río Celeste reveals a transparent appearance, indicating that the sky-blue color is due to a phenomenon involving a large portion of the water body (see box in figure 1c). Much speculation about the reasons for the characteristic sky-blue color has arisen, such as the presence of copper ions being responsible for blue tints and the dispersion of radiation generated by the presence of minerals in suspension [8]–[10]. The latter is established as the cause for most acid volcanic lakes and hydrothermal waters that present unusual water colors, such as brilliant blue, greenish blue, and cloudy emerald green [8]–[10]. Because of their striking colors, these sites are typically famous sightseeing attractions, such as Lake Goshikinuma in Japan; Lake KawahPutih in Indonesia and Yellowstone Hot Springs in USA. Their colors are due mainly to the large concentrations of mineral ions that originate from hydrothermal waters [10].

For Río Celeste, no explanation of its characteristic color has been reported. In this work, we examined the physicochemical properties of this river through field measurements, ion-exchange chromatography (IC), inductively coupled plasma atomic emission spectroscopy (ICP-OES), analysis of dynamic light scattering (DLS), X-ray diffraction and electronic microscopy. Chemical analysis showed insignificant concentration of ions such as copper, iron, or cobalt, so we eliminated the involvement of these chemical species for the sky-blue color of Río Celeste. DLS, X-ray diffraction and optical theoretical calculations demonstrated that light scattering by suspended aluminosilicate colloidal particles, under aggregation conditions, is responsible for the sky-blue of Río Celeste. The altered pH after mixing of Quebrada Agria and Río Buenavista induces the growth of suspended aluminosilicate particles, from about 184 nm to about 566 nm, with an enhanced light dependent scattering by clusters of submicron sized particles being the physical consequence of this enlargement that explains the sky-blue color.

## Materials and Methods

### Ethics statement

All necessary permits for water and sediment sampling were obtained from the National System of Conservation Areas (SINAC) of the Ministry of the Environment and Energy (MINAE) of Costa Rica (Resolution Number 052-2012-ACAT), which is responsible for the protection and management of the Arenal-Tempisque conservation area.

### Sampling and field measurements

Samples of water were collected from Río Buenavista (stream 1 in Fig. 1b), Quebrada Agria (stream 2) and Río Celeste (stream 3) in February 2no012. The temperature, pH, density, conductivity and concentration of dissolved oxygen of all streams were measured (YSI Model 85 hand-held oxygen, conductivity, salinity and temperature system) in the field. Water samples for chemical analysis, were collected from all sites using clean polyethylene bottles, chilled in ice, and stored at 4°C until their analysis. Samples for scanning electron microscopy-energy dispersive spectroscopy (SEM-EDS) were obtained from the solid material deposited at the bottom of Río Celeste (see Fig. S1). For light-scattering experiments, the samples were collected in polyethylene bottles without treatment.

### Chemical analysis

The water samples were analysed for major anions and cations as well as metal species following standardized analytical procedures at the Department of Earth Sciences of Utrecht University [16]. Samples were filtered in the laboratory with 0.45 µm polycarbonate membrane filters prior to analysis. The anions were determined by ion-exchange chromatography (IC) using a fully automated Dionex Model DX-120 system equipped with a Dionex DS4-1 autosuppresor-conductivity detector with a thermally controlled conductivity cell, a Midas Spark auto-sampler and a system controller. An anionic exchange resin Dionex IonPac analytical column (AS14, 9-µm particle size, 4×250 mm), protected with a Dionex guard column (AG14, 4×50 mm) was used. Running conditions were: 35°C, a 3.5 mM Na_2_CO_3_/1.0 mM NaHCO_3_ (pH = 10.60) mobile phase and a flow rate of 1.2 ml/min (pressure of 1632 psi). Reagents used for the mobile phase were analytical-grade. The anions were identified and quantified by comparison to certified commercial solution standard. A certified synthetic solution (Dionex 7 anion certified standard solution) was also injected for quality control and to monitor the response and reproducibility of peak areas and retention times. Repeated analysis (n = 14) of a water sample selected arbitrarily yielded relative standard deviations better than 4% for all of the anions. All standard solutions were done with ultrapure water (18.2 MΩ cm). Detector response signals were integrated using the chromatography management system Dionex PeakNet 5.1. For the ICP-OES analyses, certified multi-element stock solutions with a 5% HNO_3_ matrix and a 5% v/v HNO_3_ solution (prepared from a 65% ultrapure HNO_3_) were used to prepare standard solutions. Generally the relative standard deviation of the ICP-OES analytical determinations was within 3%.

### Dynamic light scattering and zeta potential measurements

The sizes, reported as apparent hydrodynamic diameters, and zeta potentials were recorded with a Zetasizer Nano ZS90 (Malvern Instruments Ltd., Worcestershire, UK). Samples were obtained as stated above and diluted 1∶10 to conform to the optical requirements of the instrument. Data are reported as mean ± standard deviation (*n*  =  5) at 25°C.

### Scanning Electron Microscope and Electron Dispersive Spectrometer (SEM-EDS) experiments

The chemical elements that constitute the colloidal material of Río Celeste were determined with SEM-EDS. These experiments were performed on the solid material deposited on stones in the river bed at the bottom of Río Celeste (see Fig. S1a). The white solid was dried in the laboratory near 22°C, recovered carefully with a camel-hair brush and analysed with a scanning electron microscope (Hitachi S-570) with energy-dispersive X-ray spectra (SEM-EDS).

### Characterization of solid deposits by X-ray diffraction and IR spectroscopy

Representative samples of solid sediments deposited in Río Celeste bottom were characterized by X-ray powder diffraction using a D8 Advanced (Bruker) X-ray diffractometer with CuKα1/kα2 radiation. Fourier transform infrared spectroscopy was performed using a Spectrum 1000 FT spectrometer (Perkin Elmer). Powdered samples were mixed with KBr. The resulting powder was finely ground and pressed into an optically clear pellet using a hydraulic press. Spectra were collected over the range from 400 to 4000 cm^−1^.

### Calculation of light scattering

As part of the analysis to establish a relation between the apparent hydrodynamic diameter of the particles with the sky-blue color, in our model we assumed that Río Celeste waters contain only aluminosilicate particles at low concentration (1.3 mgL^−1^). This small concentration is consistent with the transparent appearance of water samples taken from the river in small containers (see box in Fig. 1c). To correlate the visual appearance of the river's surface with the presence of aluminosilicate particles randomly distributed through the water, we evaluated the cross sections and diffuse reflection spectra. The normalized dimensionless scattering and extinction cross sections were evaluated from the scattering coefficients (a_n_ and b_n_ with n = 1,2, … specifying the multipole orders considered) with these expressions,
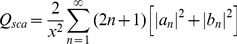
(1a)

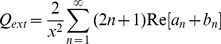
(1b)in which 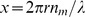 is the size parameter of the particles of radii *r*, *n_m_* is the refractive index of the medium surrounding the particles (water in our case), and λ is the wavelength of the incident radiation in vacuum. The scattering coefficients depend on the size parameter and on the wavelength dependent relative refractive index of the particles (*m = n_p_/n_m_* with *n_p_* as the particle refractive index), i.e. *a_n_ = a_n_(x,m)* and *b_n_ = b_n_(x,m)*, with explicit expressions obtained from application of appropriate boundary conditions for the electric and magnetic fields at the surface of a spherical particle [17]. The cross sections are for scattering 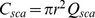, for extinction 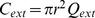, and for absorption 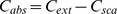. Intrinsic scattering and absorption coefficients per unit length in the medium are 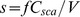 and 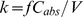 respectively, where *f* is the volume fraction occupied by the particles and V the particle volume. For each stream we associate a corresponding volume fraction f_i_ with *i = 1,2,3*. From the ratio between areas of the two peaks corresponding to particle populations in streams 1 and 3 we correlate the particle volume fraction in stream 1 with volume fraction in stream 3: 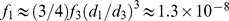, with d_1_ = 184 nm and d_3_ = 566 nm as average values of the particles diameters in the corresponding streams. Stream 2 can hence be considered to lack relevant suspended materials 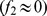. As mentioned above, we assumed that Río Celeste waters contain only aluminosilicate particles, at a concentration of approximately 1.3 mgL^−1^ which correspond to a volume fraction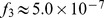. This is the order of magnitude of the volume fraction required to approach the measured direct transmission spectrum from four-flux radiative transfer calculations [18].

The dimensionless optical thickness of the medium is given by 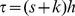 with *h* as the thickness of the medium, in our application, the average depth of the river that was determined in the field (h = 1.1 m). The wavelength dependent refractive index and extinction coefficient of water were taken from literature [19]. Considering the suspended material as submicron sized aluminosilicate particles, we approximate their optical constant using the refractive index corresponding to aluminosilicate glasses, according to the Sellmeier's relation established by Ghosh *et al*.[20]. Through the spectral range considered, the aluminosilicate particles do not display light absorption, and the small absorption by the water medium surrounding the particles has been considered in our radiative transfer calculations.

Diffuse reflection spectra corresponding to two aluminosilicate particle size distributions were evaluated from a four-flux radiative transfer model by assuming normal incidence of non-polarized collimated radiation on the front flat interface of a non-homogeneous medium [21]. The model takes into account two collimated fluxes (I_c_ and J_c_) and two diffuse ones (I_d_ and J_d_). I_c_ and J_c_ propagate in the forward direction, and I_d_ and J_d_ in the backward direction. The collimated fluxes decay with the propagation distance, due to scattering and absorption, according to the Beer-Lambert law, and two coupled energy balance equations determine the propagation distance dependence of the diffuse fluxes. The total reflection and transmission of light is determined by contributions from a collimated flux and a diffuse flux propagating in the backward or forward directions with respect to the incident direction. Explicit expressions for the collimated and diffuse components of reflection and transmission were obtained on applying boundary conditions at the flat interfaces of the non-homogenous medium [18]. The dynamic light scattering (DLS) analysis provided background information about the size of the particles in each of the streams involved. Also the corresponding size dispersions were considered in the calculations. These data have been considered to evaluate from the Mie theory the scattering cross sections of single spherical particles, and from a generalized Mie theory the scattering cross sections of clusters of aluminosilicate particles. To this extent, we designed clusters with 8 aluminosilicate particles, which present hydrodynamics radii close to that measured for the aluminosilicate aggregates in Río Celeste stream. In this way, the effect of aggregations is clearly displayed. From the reflection spectrum corresponding to the agglomerated aluminosilicate particles in water, the chromaticity coordinates (x,y) were evaluated on assuming solar spectral irradiance AM1.5 [22], and on using the color-matching functions specified by the international Commission on Illumination (CIE: Commission Internationale de l’Eclairage) [23].

## Results and Discussion

### Physicochemical analysis indicates that the sky-blue color of Río Celeste is not due to cationic species

As mentioned above, the color of Río Celeste is produced after the confluence of Quebrada Agria (Fig. 1b, stream 2) that is fairly acidic (pH 3.1, temperature 23.9°C) and Río Buenavista (Fig. 1b, stream 1) that is near neutral (pH 6.8, temperature 20.8°C) (table 1). The mixture of these streams generated the appearance of the sky-blue color (Figs. 1b and 1c). About 5 m downstream from the location of the mixing, the pH is 5.0 and temperature 22.0°C (Fig. 1b, stream 3, see also table 1). One hypothesis proposed to explain the intense sky-blue hue of Río Celeste is the presence of metallic ions such as copper. Diverse metals might be found in rivers from natural sources in which metal ores are present in the rocks over which the river flows or in the aquifers feeding water into the river [24], [25]. To analyze whether the presence of metallic ions can cause the color of the river, we quantified the chemical content at the sampling points with IC and ICP-OES. The results of chemical analysis only reflect the composition of dissolved species in the water samples and do not take into account the composition of particulate materials, due to the fact that no chemical digestion process was previously applied. As shown in table 2 minute levels of metals such as copper, cobalt or nickel were detected in all streams, indicating that the sky-blue color is not due to the presence of these chemical species. The concentrations of other colorless constituents such as Zn, Pb, As, and Cd are not remarkable. Furthermore, the results in table 2 reveal that Quebrada Agria is enriched in minerals such as sulfate (190 mg L^−1^), chloride (71 mg L^−1^), calcium (55 mg L^−1^) and silicon (38 mg L^−1^), whereas Río Buenavista contains silicon as the main component (25 mg L^−1^).

**Table 1:** Physicochemical properties of Río Buenavista, Quebrada Agria and Río Celeste.

Properties	Quebrada Agria	Río Buenavista	Río Celeste
temperature /°C	23.9	20.8	22.0
pH	3.1	6.8	5.0
conductivity /µS	691	152	309
total solids /ppt	0.3	0.1	0.2
density /g mL^−1^	1.004	1.003	1.004
dissolved O_2_/mg L^−1^	9.10	10.90	9.15

**Table 2:** Chemical composition/ppm of Río Buenavista, Quebrada Agria and Río Celeste.

Element/ion	Quebrada Agria (mg L^−1^)	Río Buenavista (mg L^−1^)	Río Celeste (mg L^−1^)
sulphate	190	13	104
chloride	71	10	31
fluoride	1.0	0.20	0.65
sodium	14	7.0	10
potassium	3.9	2.7	2.2
calcium	55	17	32
magnesium	11	5	7
aluminium	12	ND	5.0
boron	1.0	ND	0.57
silicon	38	25	29
strontium	0.18	0.11	0.13
arsenic	ND	ND	ND
iron	ND	ND	ND
nickel	ND	ND	ND
cobalt	ND	ND	ND
copper	ND	ND	ND
zinc	0.03	ND	ND
cadmium	ND	ND	ND
chromium	ND	ND	ND
manganese	0.53	0.02	0.28
lead	ND	ND	ND

ND =  Not detectable.

Relative standard deviations for the IC and the ICP-OES analyses were better than 4% for all of the anions and within 3% for all of the cations.

Río Celeste contains intermediate concentrations of all these chemical species: sulfate (104 mg L^−1^), chloride (31 mg L^−1^), calcium (32 mg L^−1^), and silicon (29 mg L^−1^). Although chemical analyses failed to reveal the presence of a metallic ion that can contribute to the color of the river, these analyses indicate the presence of sulfates and silicates at significant concentrations.

### SEM-SDS, X-ray diffraction and DLS experiments revealed the presence of colloidal material suspended in Río Celeste

Ohsawa *et al*. [10] showed that the observed blue-green color in hydrothermal ponds and bathing pools in countries such as Japan, New Zealand and China is caused by the scattering of sunlight by aqueous colloidal silica particles. Those authors suggested that aqueous silica colloidal particles of sizes between 0.1 and 0.45 µm cause the blue color of the thermal waters by Rayleigh scattering, and that particles of size greater than 0.45 µm makes the thermal water milky white by Mie scattering [10]. Onda *et al*. [26] who investigated the cloudy emerald-green hue of Yugama Crater Lake on Mount Shirane, Japan, reported that the water color is due to an optical interaction involving Rayleigh and Mie scattering by colloidal sulfur particles, causing blue and cloudy white colors, respectively, and absorption by dissolved iron (II) ions cause a green color [9], [26].

**Figure 2:**
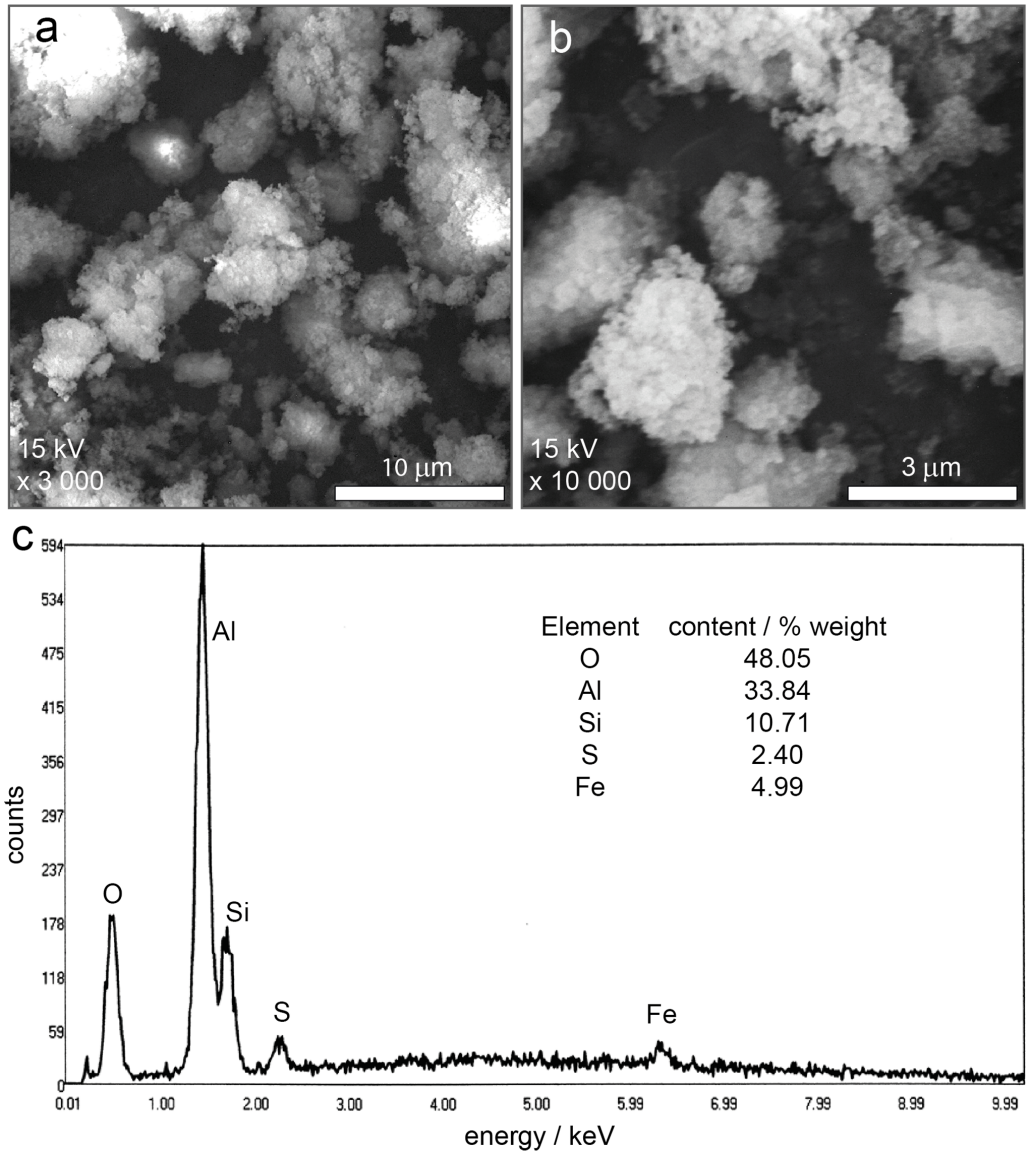
SEM-EDS analysis of Río Celeste sediments. (**a**) **and** (**b**) SEM micrographs to 10 µm (15kV×3,000) and 3 µm (15kV×10,000) respectively of the pale yellow powder sedimented at the bottom of Río Celeste. Micrographs indicate that the material is formed by an agglomeration of smaller particles deposited successively at the bottom river. (**c**) EDS analysis of the pale yellow powder showed that it is composed mainly of aluminium, silicon and oxygen. Other species such as iron and sulfur were also found in the sediment in minor proportions.

To investigate whether similar phenomena are applicable in Río Celeste, we devised pertinent experiments. The first evidence is the presence of much particulate material precipitated at the dye point (and along much of the stream; see Figs. 1b and 1c), forming a pale yellow powder as sediment in the riverbed. SEM micrographs of these sediments (Fig. 2a and 2b) show that the pale yellow powder results from the agglomeration of smaller particles that are deposited successively on the bottom of the river. A diffractogram of this sedimented powder (see Fig. S1) indicates an amorphous morphology (no sharp features), and also shows evidence of the presence of aluminosilicates through the broad line in interval 20°–35° [27]. The infrared spectra of the sediments also confirm the presence of aluminosilicates (see Fig. S1) [28], [29].

A SEM-EDS spectrum (Fig. 2c) of the particulate material demonstrates the content of silicon (10.71%), aluminium (33.84%) and oxygen (48.05%) as main elemental components. These concentrations strongly suggest the presence of Al_2_SiO_5_ aluminisilicates. Other species such as iron (4.99%) and sulfur (2.40%) were also found in minor proportions in the solid (Fig. 2c). These mineral sediments appear to have originated from the precipitation of suspended particles and hence that the chemical nature of both is similar; a distinction is that the particles in the sediment are larger than those in aqueous suspension. All data indicate the presence in Río Celeste of suspended material formed by amorphous aluminosilicates and other minerals such as iron oxides in a mixture. We observed the presence of some particles that were attracted with a magnet; that magnetic property provides also evidence of the content of iron oxides in the sedimented material [30], [31].

**Figure 3:**
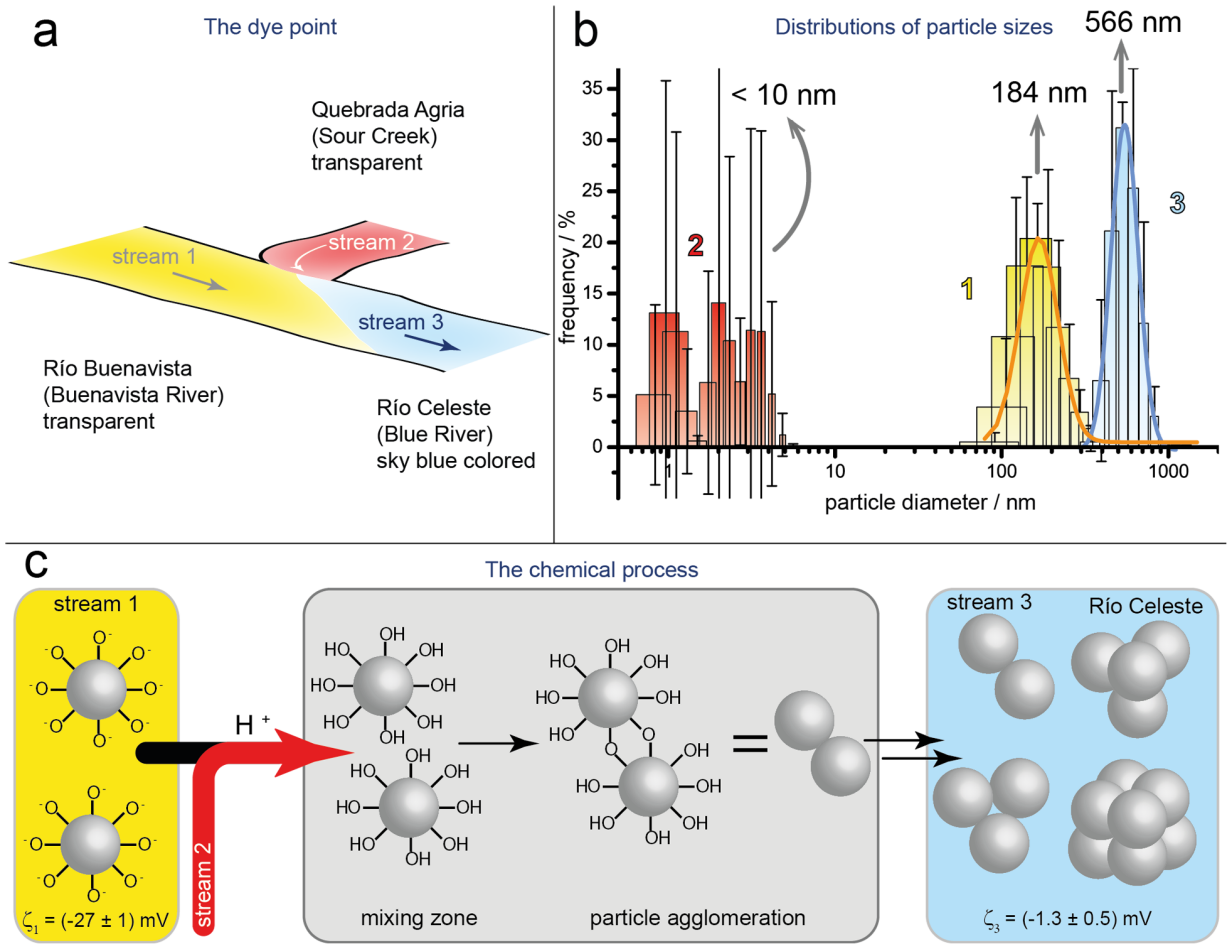
Dynamic light scattering experiments and the mechanism of enlargement of colloidal particles. (**a**) Sketch of the confluence of Río Buenavista (stream 1, yellow) and Quebrada Agria (stream 2, red). Mixing of the two streams produces the appearance of the sky-blue color (stream 3, Río Celeste in blue). (**b**) Hydrodynamic diameter distribution of particles (measured by intensity) in the suspended material in the three streams. Colors and numbers represent the positions in Fig. 3a. (**c**) The chemical mechanism of enlarged particles after mixing of the water streams at the dye point (“Teñidero”).

Based on these results and the reports of explanations of the blue color of thermal waters [8], [10], [26], we performed experiments of dynamic light scattering to detect suspended solid materials on samples of water from the colorless streams of Río Buenavista (stream 1, see Figs. 1b and 3a) and Quebrada Agria (stream 2) and from the mixed sky-blue stream of Río Celeste (stream 3). This DLS technique enabled an extraction of the size distribution of suspended solid materials in the water samples (Fig. 3b). The data show that the suspended materials in stream 2 are small (<10 nm in diameter) and almost randomly distributed; this stream can hence be considered to lack relevant suspended materials. In contrast, the transparent water sample from stream 1 showed sizes with a log-normal distribution about diameter 184 nm for solid particles in suspension, and the blue water of stream 3 showed sizes with a log-normal distribution of the suspended particulate material with a central value 566 nm.

To explain the increased size of particles in suspension after mixing of the water streams, we combine our experimental evidence and postulate a chemical mechanism for the enlargement of the particulate material (Fig. 3c). The DLS experiments indicate that the particulate solid materials in stream 2 are so small that it is reasonable to state that the particulate material arises from the transparent stream 1. These two streams show disparate pH: stream 1 is nearly neutral and stream 2 is acidic (see table 1); the mixed streams present an intermediate pH = 5.0. At the dye point, the decreased pH favours the protonation of aluminosilicate particles from stream 1 (Fig. 3c), which yields reactive hydroxyl groups at the particle surfaces. The nearly neutral surface of the resulting protonated particles causes a suppression of the electrostatic repulsion between particles, producing their agglomeration and sedimentation. These facts provide a mechanism for the enlargement of the suspended particles after mixing of transparent streams 1 and 2 [32]–[35]. This mechanism is confirmed from the measurement of the zeta potential (ς)[36] of the suspended particles (Fig. 3c); this physical parameter provides a measure of the surface charge in suspended colloidal materials. The zeta potential of the small particles in stream 1 is ς_1_ =  (−27±1) mV; the corresponding value for the larger particles in stream 3 is ς_3_ =  (−1.3±0.5) mV. The decreased (in absolute value) zeta potential after mixing of the streams at the dye point is consistent with the protonation of the particles and therefore with the enlargement of the particles by agglomeration. This fact is also in accordance with the observed precipitation of particles at the dye point, and finally becoming the reason of the non-homogeneous medium of particles suspended in the water stream of the blue river.

All these data indicate that the increased size of the suspended material at the dye point is associated with the sky-blue hue, because of specific light scattering exerted by the larger particles in the water stream of the blue river. To confirm this hypothesis, we explored by means of computational calculations based on a four-flux radiative model whether or not, a non-homogeneous medium consisting of suspended particles in water with the found distribution of particle sizes at the blue river, correlates with light scattering in the blue region of the visible spectrum.

### Computational modelling based on the diameters of colloidal particles indicates that light scattering occurs in the blue region of the visible spectrum

**Figure 4:**
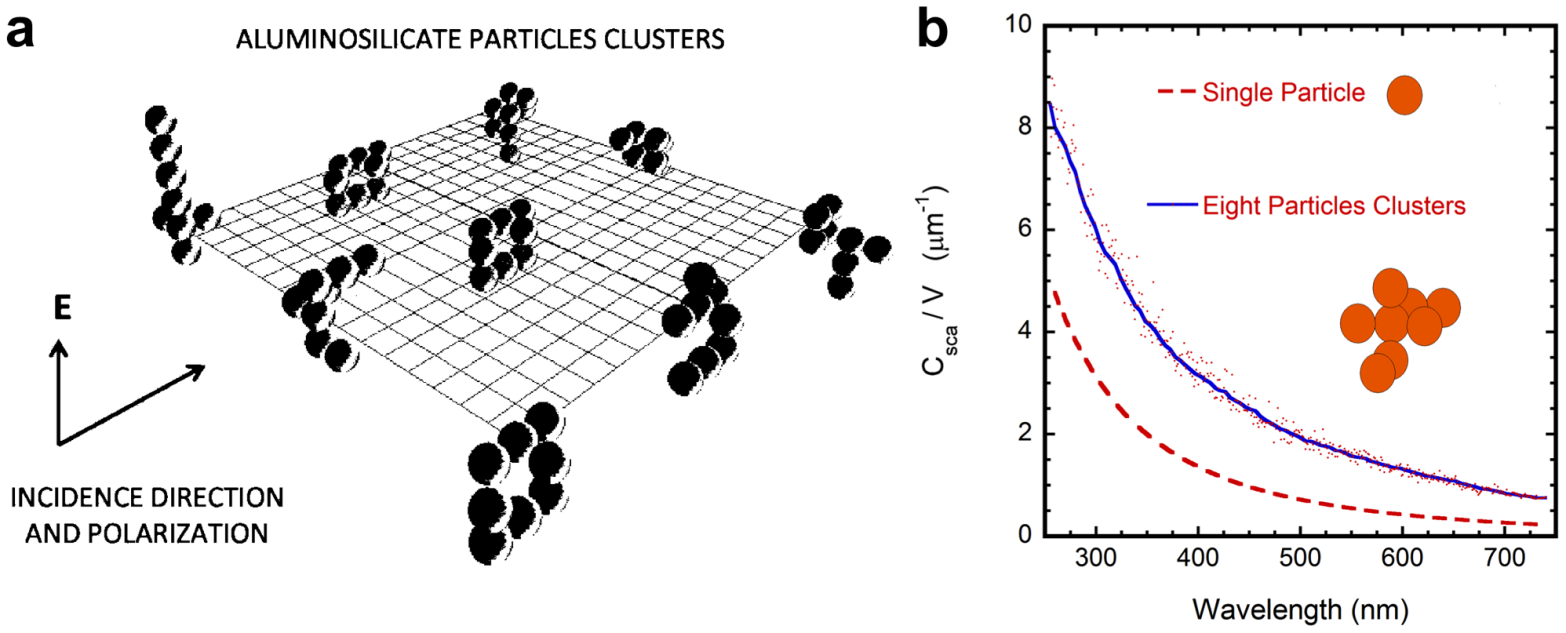
Aluminosilicate particles clusters and the average volumetric scattering cross section per particle. (**a**) Nine clusters generated by a diffusion limited aggregation scheme to evaluate the scattering cross section per unit volume and per particle corresponding to each cluster. Each cluster consists of 8 aluminosilicate particles with an average diameter of 184 nm. (**b**) Average volumetric scattering cross section of an aluminosilicate spherical particle in an 8-particle cluster. The average diameter of the particles is 184 nm, and a log normal size distribution was considered (single particle diameters between 130 and 240 nm). The orange dashed line corresponds to the isolated single particle, and the blue solid line is obtained from averaging the calculations at each wavelength (dots) carried out for the nine clusters displayed in figure 4a.

To correlate the visual appearance of the river with the presence of suspended particles distributed through the water, we evaluated cross sections and diffuse reflection spectra. For the aluminosilicate particles, with a log normal size distribution characterized by a mean diameter d_1_ = 184 nm and a standard deviation Δd_1_ = 55 nm, average values of the volumetric scattering cross section (*C*_sca_/*V*) were evaluated with the Mie theory when considering single isolated particles, and from the generalized Mie theory of Gerárdy and Ausloos (by assuming a quadrupole approximation in the expansions of the fields) in the case of aggregated or agglomerated particles [37]–[40]. The relative error of this quadrupole approximation, respect to the next octupole one is between 15% (in the ultraviolet) and 1% (in the red side of the visible spectrum). The average relative error in the visible range is close to 3%, decreasing from 7% at short wavelengths to 1% for large ones. The agglomeration of the 184 nm sized particles in clusters was simulated by application of a diffusion limited aggregation scheme [41]. Clusters with 8 particles are characterized by hydrodynamics radii close to that measured for aluminosilicates in Río Celeste stream (i.e 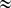
566 nm), with our evaluation of this radius based on an average of the gyration radius and the radius of a sphere with the same volume occupied by the cluster. The results are depicted in Fig. 4 where we also display the geometric arrangement of nine clusters (Fig. 4a) whose scattering cross sections were evaluated to obtain an average value per particle at each wavelength considered. The more significant effect of aluminosilicate particles aggregation is to increase, in an average sense, the scattering cross section per unit volume and per particle. With respect to an isolated sphere, the average volumetric scattering cross section of a similar particle in a cluster is around twice that of the isolated one (Fig. 4b).

**Figure 5:**
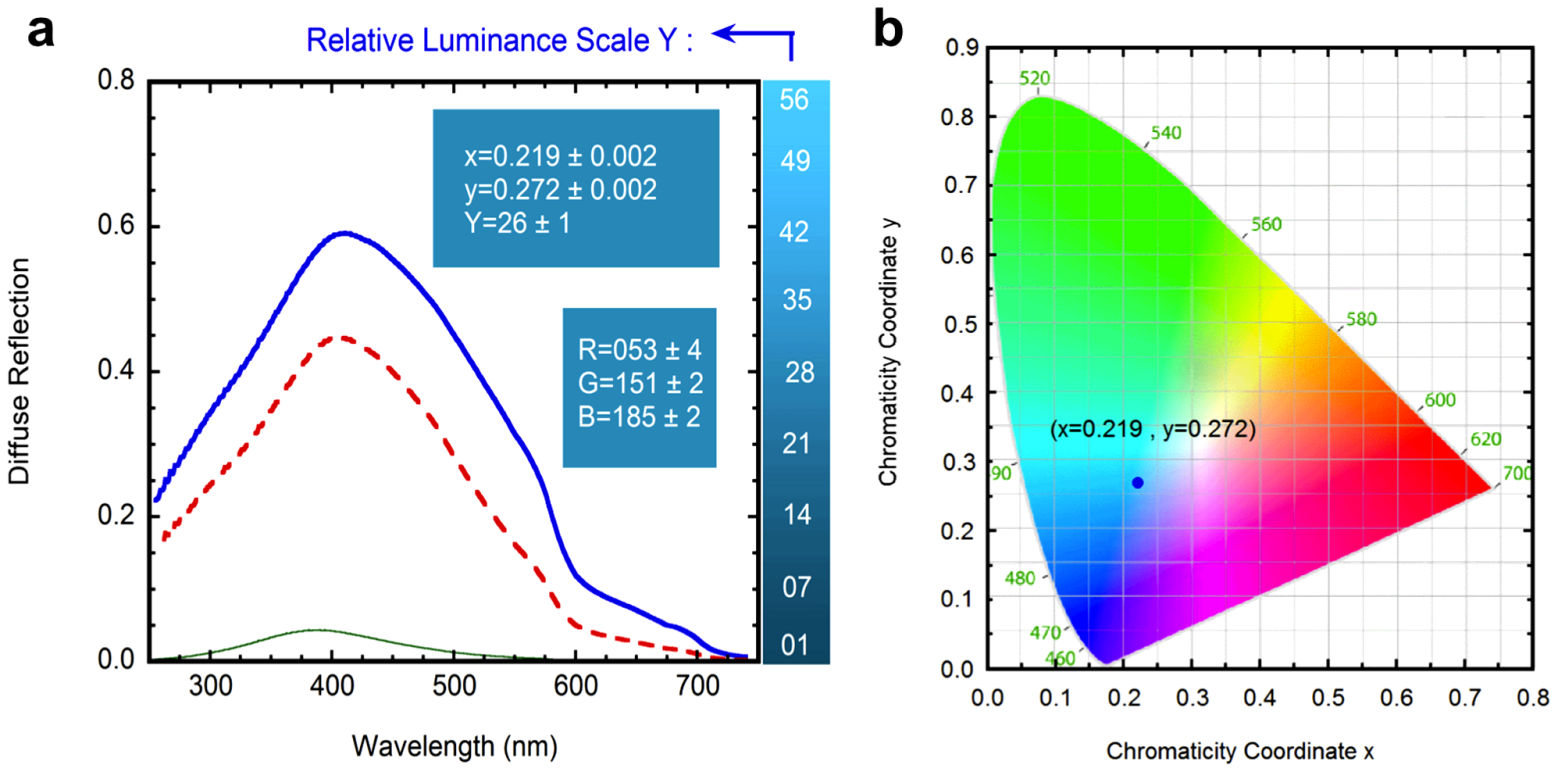
Computational modelling of light scattering. (a) Diffuse reflection spectra of aluminosilicate particles in water for aggregated (blue solid line) particles, and for non-aggregated aluminosilicates (red dashed line) at a volume fraction 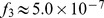. The solid green thin line, displaying low reflection values, corresponds to non-aggregated aluminosilicate particles at a volume fraction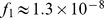. The insets show the chromaticity coordinates corresponding to both reflection spectra. (**b**) Chromaticity diagram indicating the coordinates of the color corresponding to the diffuse reflection spectra previously shown by the blue solid line.

Figure 5a depicts diffuse reflection spectra (R_cd_(λ)) evaluated from a four-flux radiative transfer model [18]. The three spectra have been calculated by assuming polydispersions of aluminosilicate particles in water whose average size is 184 nm. For the green solid line, evaluated from Mie theory and displaying very low reflection values, the volume fraction occupied by the particles is f_1_. This reflection spectrum is correlated with the visual appearance of the transparent Río Buenavista. The dashed red line, also evaluated from the Mie theory, corresponds to particles with no aggregation (184 nm) and occupying a volume fraction f_3_. The solid blue line displays the reflection spectrum calculated from the generalized Mie theory and by assuming aggregation of the aluminosilicate particles in clusters whose average size is 566 nm, and f_3_ being the volume fraction occupied by the particles. The three spectra show broad maxima around 400 nm, and the differences in reflection magnitudes through the spectral range arise from the two different concentrations considered (f_1_ and f_3_) and from the state of aggregation or no aggregation assumed. It can be concluded that the bluish color of Río Celeste is mainly due to the scattering of light by aggregated submicron size aluminosilicate particles, with some residual contribution of non-aggregated ones. Fig. 5a also indicates the chromaticity coordinates and respective values of the RGB parameters corresponding to the diffuse reflection spectra depicted by the blue solid line. The first ones are evaluated from the relations 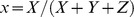, 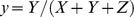, and 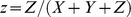 with
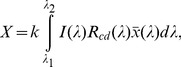
(2)where I(λ) is the AM1.5 solar spectrum [22], 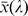 is a CIE (Commission Internationale de l’Eclairage) color-matching function, and with similar expressions for Y and Z stimulus in terms of the other color matching functions: 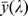 and 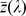 respectively [23]. For a specific color, the chromaticity coordinates (x, y, and z) correspond very approximately to the relative contributions of the red, green, and blue basic colors. In fact, x+y+z = 1 which means that only two chromaticity coordinates are required to specify a color. Through our implementation, the spectral range runs from 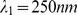 to 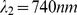. Within this framework, the stimulus Y is proportional to the luminance of a color matched by the amounts of R (red), G (green), and B (blue) contributions. The constant k in Eq. 2 is fixed by normalizing Y = 100 to a perfect reflector of the light incoming from the illuminant considered. On the right side of Fig. 5a we display a luminance bar which shows how the bluish water of Río Celeste, under different light intensity of the illumination conditions, would look. Figure 5 also displays the RGB values, chromaticity coordinates (x,y), and the relative luminance value Y, with the corresponding uncertainties arising from the fact of computing scattering cross sections within a quadrupole approximation. Finally, Fig. 5b displays the position of the chromaticity coordinates in the CIE diagram. As seen, the predicted point (values **x** = 0.219 and **y** = 0.272 in the CIE diagram) is within the blue region of the visible spectrum. Thus, the agreement of Río Celeste with a bluish coloration is clearly established.

According to the combined experimental results and computational calculations, we conclude that the characteristic sky-blue color of Río Celeste water is due to the presence of aluminosilicate particles with different degrees of aggregation (with a log-normal size distribution with a central value of 566 nm) that scatter the sunlight. Finally, as mentioned in the Introduction section, there are also areas with a milky white color, which is probably due to the precipitation of particles in suspension as seen in the dye point (Fig. 1b). After 14 km, possibly most of the material in suspension has precipitated generating the loss of blue color.

### Conclusions

Our data are consistent with the notion that Mie scattering of sunlight by aqueous colloidal aluminosilicate particles is responsible for the intense sky-blue color of Río celeste. A remarkable distinction with regard to the origin of the color of Río Celeste with respect to other thermal waters is that this blue color is produced on the mixing of two transparent streams at a point known as “Teñidero” (dye point). The agglomeration of small aluminosilicate particles due to a pH change, after mixing of the streams, causes a size increase of the suspended material up to about 566 nm in average. These submicron sized aluminosilicate particles consisting of aggregates of smaller ones leads to an enhancement of the average scattering efficiency giving a more intense bluish color for the Río Celeste stream.

## Supporting Information

Figure S1 **X-ray diffraction and IR characterization of Río Celeste sediments.** (**a**) Photography of a rock obtained from Río Celeste bottom with deposited sediments on its surface. (**b**) X-ray diffraction pattern of white sediments showed in Fig. S1a. (**c**) Infrared spectrum and characteristic IR vibrational bands of the white solid power.
(TIF)
