# Supplementary figures and images for: Scattering of Light by Colloidal Aluminosilicate Particles Produces the Unusual Sky-Blue Color of Río Celeste (Tenorio Volcano Complex, Costa Rica)
Source: PLoS One. 2013 Sep 18;8(9):e75165. doi: 10.1371/journal.pone.0075165 (PMC3776737; doi:10.1371/journal.pone.0075165)

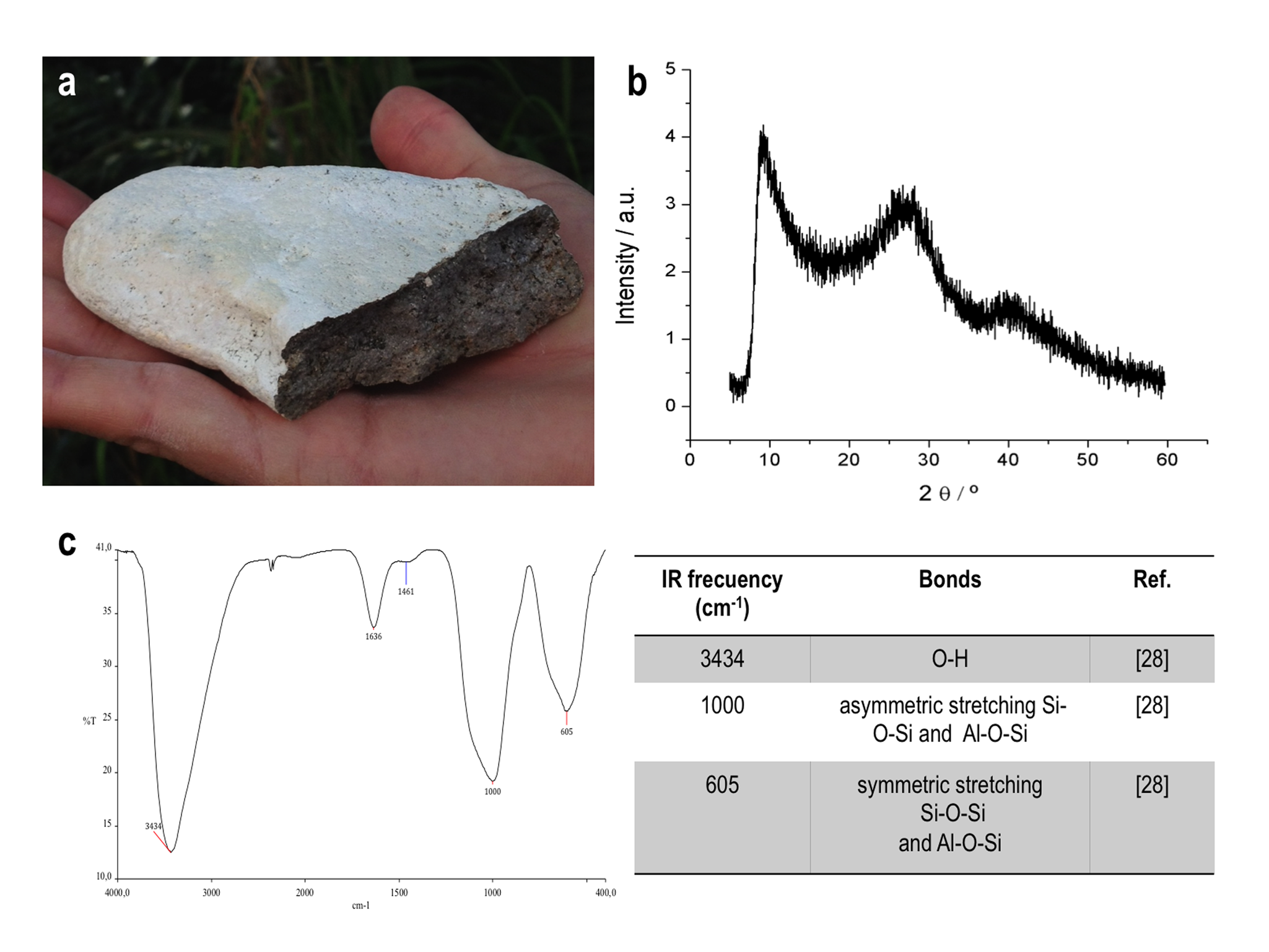

Supplement: Figure S1 — X-ray diffraction and IR characterization of Río Celeste sediments. (a) Photography of a rock obtained from Río Celeste bottom with deposited sediments on its surface. (b) X-ray diffraction pattern of white sediments showed in Fig. S1a. (c) Infrared spectrum and characteristic IR vibrational bands of the white solid power. (TIF) [file pone.0075165.s001.tif]
